# Non-Propagating Cracks at the Fatigue Limit of Notches: An Analysis of Notch Sensitivity and Size Effect

**DOI:** 10.3390/ma17184632

**Published:** 2024-09-21

**Authors:** Mirco Daniel Chapetti

**Affiliations:** Laboratory of Experimental Mechanics, INTEMA, National University of Mar del Plata—CONICET, Av. Colón 10.850, Mar del Plata B7600, Argentina; mchapetti@fi.mdp.edu.ar

**Keywords:** fatigue notch sensitivity, size effect, fracture mechanics, fatigue limit estimation

## Abstract

The issues of the high-cycle fatigue resistance of notches and the role of non-propagating short cracks in defining the fatigue notch sensitivity and fatigue limit of the configuration are addressed. A fracture mechanics approach is employed to determine the threshold configuration that defines the associated fatigue limit. The influence of notch sharpness, notch size, intrinsic fatigue limit, microstructural dimensions, and the threshold for crack propagation is examined. A simple expression is proposed to estimate the maximum fatigue notch factor, *k*_f_^Max^, which incorporates the influence of these non-propagating cracks. The fatigue limits for both blunt and sharp elliptical notches are analyzed and predicted based on experimental results reported in the literature. Additionally, shallow notches or small defects are analyzed, where it is found that the same hypothesis may not be applicable.

## 1. Introduction

When components with notches or holes are tested under cyclic loading and their fatigue strengths are measured, two key observations are typically made [1,2,3,4,5,6,7]. First, the fatigue notch factor (*k*_f_), which is the ratio of the fatigue strength of an unnotched specimen to that of a notched specimen, is generally less than the stress concentration factor (*k*_t_), defined as the ratio of the maximum stress generated by the stress concentrator to the nominal stress applied to the component. Second, the value of *k*_f_ decreases as the size of the notch or hole decreases, a phenomenon known as the notch size effect. On the other hand, in many applications, it is challenging to define a specific notch size, and the stress concentration and its influence on fatigue strength are determined by macro- and/or micro-geometrical singularities. Furthermore, there are considerable differences in the behavior of relatively large notches (greater than a millimeter in depth or radius) compared to very shallow notches or concentrators, which are on the order of the microstructural size [8].

The fatigue notch factor (*k*_f_) is influenced by both the notch geometry and the material properties, and many interpretations of the *k*_f_ < *k*_t_ effect has been given. The most popular and traditional interpretation is based on the concept of a critical distance or process zone volume [9,10,11]. According to this concept, the material is not sensitive to the peak stress at the surface but rather to an average stress over a finite volume of material that participates in the fatigue damage process.

More recently, several fracture mechanics-based approaches have been developed using the same concept, such as the critical distance/line/volume methods proposed by Taylor [12]. For example, in the critical distance method, the material parameter is estimated from the plain fatigue limit and the threshold for fatigue propagation of long cracks. While this method has been very successful in practical applications [13], it does not explicitly account for the physical mechanisms of crack initiation and short crack propagation. 

Additionally, classical Linear Elastic Fracture Mechanics (LEFM) analyses of crack propagation conditions [14,15,16,17,18] have highlighted that the presence of non-propagating cracks can significantly contribute to the *k*_f_ < *k*_t_ effect. A significant advancement in understanding why *k*_f_ is less than *k*_t_ and in quantifying fatigue notch sensitivity was achieved by El Haddad et al. [4], Tanaka et al. [2,17], McEvily and Minakawa [16], Chapetti et al. [19], Smith and Miller [3], Lukás et al. [14], among others, who explored the growth behavior of small fatigue cracks using various models. However, there remain considerable discrepancies in the proposed approaches to address this issue, particularly concerning the assumptions and limitations of each method and the hypotheses for their implementation.

In this work, both the fatigue notch sensitivity and size effect are analyzed in an integrated manner, examining their relationship to notch dimensions and intrinsic material strengths (intrinsic fatigue limit and crack propagation threshold as a function of crack length). Fracture and continuum mechanics models are used to study notches with aspect ratios less than or equal to one (semi-circular or semi-elliptical). In particular, emphasis is placed on the analysis of the occurrence and length of non-propagating cracks in both the blunt and sharp notch regimes. The method for obtaining a maximum *k*_f_ associated with a given notch depth, and therefore the minimum associated fatigue limit, as well as the corresponding non-propagating crack length, is also analyzed. Finally, the hypothesis used to apply fracture mechanics models regarding whether or not to incorporate notch depth into the development of the propagation threshold is analyzed. This hypothesis appears to be acceptable for relatively large notches but not for small notches or volumetric surface defects.

## 2. Notch Sensitivity and Non-Propagating Cracks

**Figure 1:**
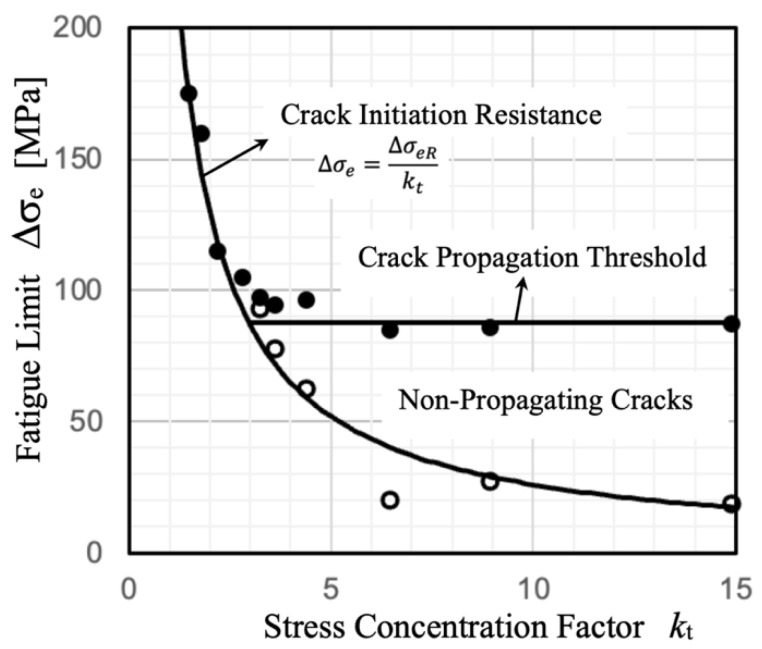
Frost diagram of fatigue limit vs. stress concentration factor, *k*_t_. Mild steel specimens having V-notches 1.3 mm deep and root radii varying from 0.005 mm to 0.3 mm [20]. Hollow circles represent the crack initiation limit, while solid circles indicate the fatigue limit (fracture).

As depicted in the Frost diagram (Figure 1) [20], there exists a critical *k*_t_ value beyond which the fatigue limit is governed by crack propagation (in the sharp notch regime). The fatigue notch factor *k*_f_ is lower than *k*_t_ because the stress required for crack propagation (around 90 MPa; depicted by full symbols in Figure 1) exceeds the stress required for crack initiation (260 MPa/*k*_t_; depicted by open symbols in Figure 1). The Frost diagram illustrates a region where non-propagating cracks occur in sharp notches. These cracks initiate under a specific applied nominal stress range but lack sufficient driving force to propagate until failure.

However, it is now well known that even in the blunt notch region, the fatigue limit can be determined by a threshold condition associated with microstructurally short non-propagating cracks [1,15,19]. For “sharp” notches (high stress concentration factor, *k*_t_), mechanically short non-propagating cracks exist at the fatigue limit of the notched component, whereas “blunt” notches (small *k*_t_) exhibit microstructurally short non-propagating cracks. In the latter case, the driving force sufficient to initiate a crack at the notch root and overcome the strongest microstructural barrier defining the intrinsic fatigue limit is also adequate to propagate the crack continuously to failure. The fatigue strength in this scenario is said to be governed by a microstructural threshold determined by a Δσ criterion, which will be shown later and can also be associated with an equivalent intrinsic threshold in terms of stress intensity factor range related to the intrinsic fatigue limit. On the other hand, in the case of sharp notches, the fatigue strength is governed by a mechanical threshold defined by a ΔK criterion, rendering it independent of the stress concentration factor *k*_t_. Here, the fatigue strength is primarily influenced by the notch depth *D* and the fatigue threshold ΔK_th_ for physically short or long cracks [4,16,21,22,23].

From the statements above, a clear transition in the mechanism and the associated controlled parameters defining the fatigue limit of the notch is evident. This transition is not addressed by the traditional approaches mentioned in the previous section, which require material parameters that must be experimentally determined for a specific material and are dependent on the notch geometry. They can only account for notch sensitivity in sharp notches at a given notch depth *D*. If the geometrical parameter *D* changes, the material parameter of the model must also change accordingly. 

The existing knowledge of threshold conditions governing the fatigue resistance of various notch configurations, coupled with applicable models capable of accurately estimating fatigue crack propagation thresholds for crack lengths exceeding microstructural dimensions (e.g., grain size), provides the means to address threshold conditions associated with any geometrical and loading configuration for a given material. Models available in the literature can estimate configurations of fatigue crack propagation thresholds and associated non-propagating crack lengths with acceptable accuracy [4,16,17,24]. Previous studies demonstrate the feasibility of estimating fatigue notch sensitivity using fracture mechanics-based approaches [2,3,14,16,17,18,19,23]. However, the transition between conditions still cannot be adequately addressed in a continuous and integrated manner, necessitating separate consideration of the size effect, particularly for sharp notches, using a complementary approach or model.

More recently, additional analyses have been conducted and attempts have been made to progress towards a comprehensive clarification of these issues [25,26,27]. However, as will be seen later, some aspects still need clarification, particularly concerning the formulation of hypotheses and limitations of model application.

Next, the sensitivity to the blunt notch is analyzed, focusing on the intrinsic fatigue limit and the position of the microstructural barrier that defines it. Then, the comprehensive analysis using the fracture mechanics approach is discussed, including how notch size can be incorporated into the analysis to infer the transition between blunt and sharp notches, considering both the stress concentration effect and the size effect.

## 3. Threshold Conditions for Plain and Notched Specimens

In the current understanding, the high-cycle intrinsic fatigue limit is no longer considered a critical stress for crack initiation. Instead, it signifies the stress level below which an already initiated micro-crack cannot propagate. Essentially, the intrinsic fatigue limit serves as a threshold stress for micro-crack growth. This concept was clearly articulated by Miller in reference [1], using a Kitagawa–Takahashi diagram [28] that illustrates the threshold stress range for crack propagation as a function of crack length. This limit is material-specific and depends on the microstructural characteristic dimension, *d* [1]. Early support for this concept can also be found in the comprehensive analyses of Tanaka et al. [15] and Tokaji et al. [29].

**Figure 2:**
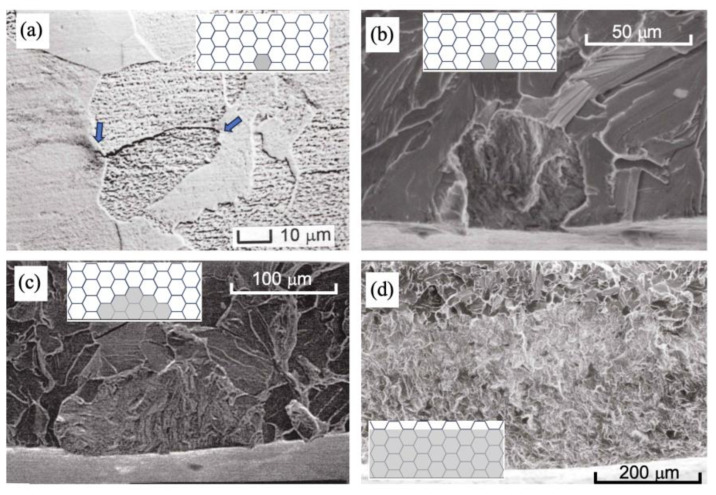
(**a**–**d**) Examples of experimentally observed non-propagating cracks in ferritic carbon steel samples with stress concentrators. Schematics illustrating the arrangement of cracks relative to grains are also provided for each case. *d* = 0.055 mm. Arrows indicate surface crack tips.

Previous research by Kitano, Chapetti, and their colleagues [19,30,31,32] explored the position and the effective resistance of microstructural barriers, establishing their relation to the intrinsic fatigue limit. These studies provided additional evidence that the intrinsic fatigue limit for plain and blunt-notched samples is defined by the strongest microstructural barriers. In those works, the position and the effective resistance of microstructural barriers were analyzed in relation to the fatigue limit of semi-circular blunt-notched specimens (*k*_t_ = 1.46, 1.94, and 2.51, for notch depths *D* = 1 mm and root radii ρ = 2, 1, and 0.5 mm) across various low-carbon steel microstructures (ferrite, ferrite–bainite, and bainite–martensite). Following machining, the notches were polished and chemically etched, allowing observation and analysis of non-propagating cracks that nucleated at stress levels just below or at the fatigue limit (runout of 10^7^ cycles), using a scanning electron microscope. In the complementary analysis conducted by Kitano et al. [30] and Chapetti et al. [31] on low-carbon steels with similar microstructures dimension *d* (ferrite), and using three stress concentrators (*k*_t_ = 1.46, 1.94, and 2.51), non-propagating crack lengths ranged from the microstructural size *d* (e.g., ferritic grain size *d* = 0.055 mm) to approximately 0.250 mm (five times the grain size *d*). As the stress concentration factor *k*_t_ increased, the non-propagating crack length increased discretely, determined by the distribution of microstructural barriers located at average distances from the surface: *d*, 2*d*, 3*d*, and so forth. Figure 2 provides additional examples from these studies on observed non-propagating cracks in a ferrite microstructure. 

**Figure 3:**
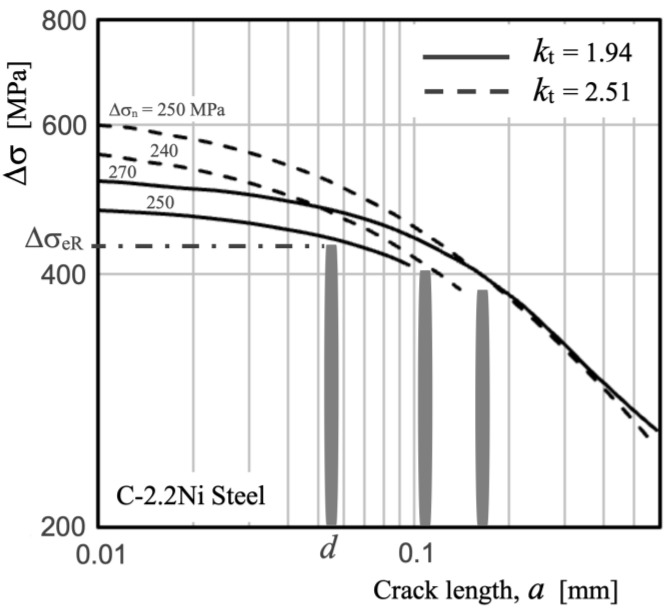
K–T diagram. Example of the influence of the microstructural barrier in the definition of the blunt notch sensitivity for a ferrite steel with intrinsic fatigue limit Δσ_eR_ = 430 MPa and average ferrite grain size *d* = 0.055 mm. The position and resistance of the microstructural barriers and applied stress distribution for *k*_t_ = 1.94 and 2.51 are shown. Nominal applied stress values are indicated.

Figure 3 depicts the Kitagawa–Takahashi diagram illustrating the stress distribution from the notch root surface for two different notches (*k*_t_ = 1.94 and 2.51), along with the estimated position and resistance of the first three barriers in a ferritic microstructure with an average grain size of *d* = 0.055 mm (0.1% C, 2.2% Ni carbon steel, yield and tensile strength of 284 and 438 MPa, respectively, intrinsic fatigue limit Δσ_eR_ = 430 MPa for *R* = −1, H_V_ = 140 [31]). In the analysis, an average value of *d* is assumed, acknowledging that while the size of microstructural entities has an associated dispersion, as the crack grows, it traverses a greater number of ferritic grains, and its length is approximately determined by a certain average number of microstructural entities.

In the case of an unnotched specimen subjected to uniaxial loading, the stress distribution would be constant, and the intrinsic fatigue limit is determined by the strongest microstructural barrier, given in this case by the first grain boundary located at an average distance *d* from the surface. As the stress concentration factor increases, stress gradients develop that allow the initial barriers to be overcome and induce crack arrest at greater depths due to deeper barriers (located at distances *d*, 2*d*, 3*d*, … from the surface). On the other hand, the concentration effect results in stress levels near the surface being similar or somewhat higher than the intrinsic fatigue limit under lower nominal stresses. Thus, the fatigue limit Δσ_e_ decreases to 270 MPa for *k*_t_ = 1.94, and to 250 MPa for *k*_t_ = 2.51, due to crack arrest at deeper microstructural barriers, as shown in Figure 3.

**Figure 4:**
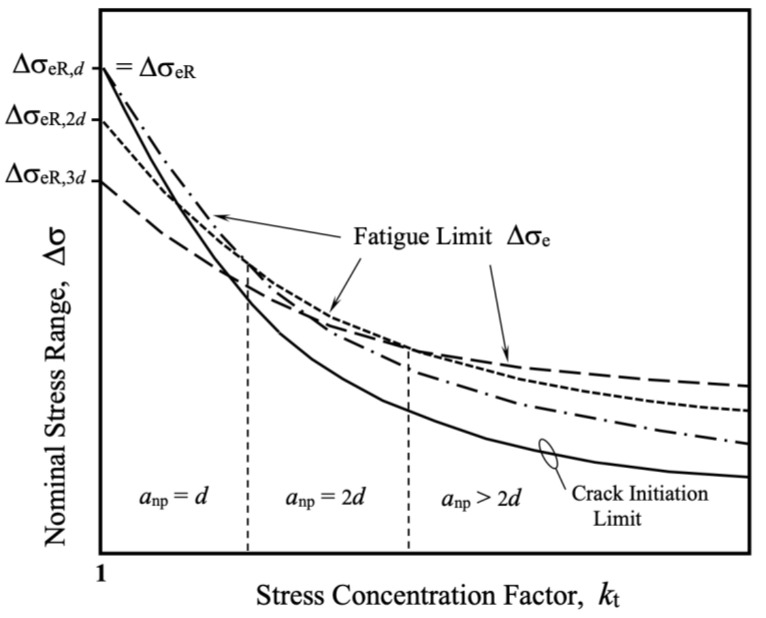
The fatigue limit Δσ_e_ of blunt notches defined as the greatest fatigue limit associated with the effective resistance Δσ_eR,nd_ and the position from the notch-root surface *nd* of the microstructural barrier *n*.

As a result of the previous experimental evidence (Figure 2 and Figure 3), an intrinsic fatigue strength Δσ_eR,nd_ can be defined for each barrier located at a given distance *nd* from the surface. For each stress concentration factor *k*_t_ associated with a stress concentrator, each microstructural barrier, characterized by its strength and position relative to the surface, will define a specific fatigue strength. The fatigue limit Δσ_e_ of the notch will be determined by the highest strength associated with the different microstructural barriers. The length of the non-propagating crack associated with the notch will be determined by the position of the microstructural barrier defining its fatigue limit. This concept is illustrated in Figure 4, which shows the fatigue limit curves as a function of *k*_t_ for three microstructural barriers located at distances from the surface equal to *d*, 2*d*, and 3*d*, with *d* being the average microstructural size. It is important to note here that the concept used for each barrier is similar to that used in the well-known point stress approach [12], with the difference that the latter considers only one microstructural barrier to overcome for any *k*_t_.

From Figure 4, it can be inferred that each microstructural barrier will define, for a given *k*_t_, a fatigue notch factor *k*_f_ that is lower than *k*_t_. This factor *k*_f_ will clearly depend on the distance of the barrier from the surface and its strength. For blunt notches, the smaller the value of the microstructural dimension *d*, the closer the fatigue limit is to that given by Δσ_eR_/*k*_t_. 

Finally, it is important to note that experimental observations indicate that the early propagation threshold of small cracks is discontinuous and is defined by microstructural barriers spatially distributed relative to the surface. However, already at a depth equivalent to two microstructural barriers, the surface length of the crack spans at least three microstructural entities (for example, three ferritic grains in a semi-circular crack). A crack arrested by a third microstructural barrier would involve at least five entities if considering a semi-circular crack. The presence of stress gradients due to the concentrator increases the aspect ratio *a/c* (depth-to-surface semi-length ratio) as the crack depth increases. Figure 2 shows that for a stress concentration factor *k*_t_ = 2.51, the depth of the arrested crack associated with the fatigue limit corresponds to about five microstructural entities (five times the average size of the ferritic grain, *d*), and the aspect ratio has decreased to become practically zero (*a*/*c* ≈ 0, crack with a straight front).

Two very important aspects can therefore be inferred, which will serve as hypotheses when proposing any attempt to predict fatigue limit using fracture mechanics approaches: (i) the assumption that the propagation threshold can be considered a continuous function of crack depth (which implies the ability to use continuous and differentiable equations); and (ii) that in the case of notches, the propagation threshold (in terms of stress range, Δσ_th_, or stress intensity factor range, ΔK_th_) must consider the transition of the crack aspect ratio *a*/*c*, which changes rapidly from *a*/*c* ≈ 1 for its arrest at the first microstructural barrier *d* (in the case where the crack naturally nucleates from the surface in a microstructural entity or a defect of similar size), to *a*/*c* ≈ 0 for a crack depth involving only five microstructural entities (or for a given distance from the notch surface). Thus, any approach for predicting the fatigue limit of sharp notches can hypothesize that the non-propagating crack considered in the analysis will have an aspect ratio *a*/*c* = 0.

Next, taking into account these and other concepts, the formulation and utilization of fracture mechanics methodologies that employ predictive models for estimating the propagation threshold as a function of crack length are analyzed. It is also necessary to understand how to estimate the applied driving force, particularly in cases where a transition is experienced between the exclusive influence of the stress field at the notch root and the range of cracks where the notch size (notch depth *D*) begins to dominate.

## 4. The Fracture Mechanics Approach

The fracture mechanics approach employed in the present study is based on the concept of the resistance curve (or alternatively the cyclic *R*-curve [17]) for fatigue crack growth [4,16,17,24,26,33,34], which is defined by the threshold for fatigue crack propagation as a function of the crack length (including the short crack regime), ΔK_th_ vs. *a*. The effective driving force for crack propagation is obtained as the difference between the total applied driving force, ΔK, and the threshold for crack growth, ΔK_th_, for any crack length. The fatigue crack growth rate is then expressed, for instance, by the following simple expression [35]:

(1) $$\frac{da}{dN}=C{\left(∆K-∆K_{th}\right)}^{m}\;$$

**Figure 5:**
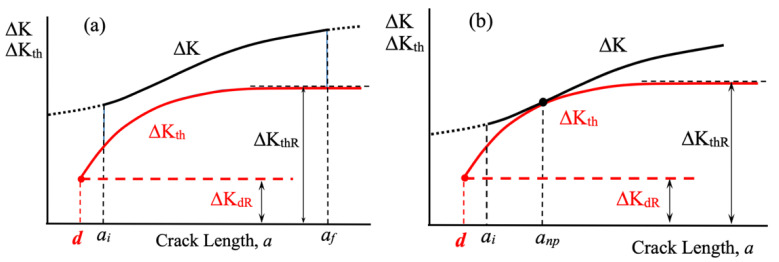
Fracture Mechanic Resistance Curve Concept. (**a**) Configuration for crack initiation and propagation until fracture. (**b**) Fatigue limit configuration.

The resistance curve concept is schematically represented in Figure 5, where both the applied ΔK and the resistance to crack propagation ΔK_th_ are shown as functions of the crack length, including the short crack regime where the threshold is developed. Figure 5a illustrates the case where the applied ΔK is higher than the fatigue threshold for crack propagation, ΔK_th_, for any crack length. In this case, fracture can occur, and fatigue life can be estimated. To estimate finite fatigue lives, Equation (1) should be used, and the parameters *C* and *m* must be known for the material where the crack is likely to propagate, as well as the initial and final crack lengths.

Instead, Figure 5b shows the case where the fatigue limit of the configuration is defined by the nominal applied strength range Δσ_e_, for which the applied ΔK becomes equal to the threshold ΔK_th_ for a given crack length, representing the associated non-propagating crack length. The maximum allowable defect size for that configuration can also be determined, allowing for the analysis of the defect size sensitivity of the configuration.

The analysis of the applied ΔK and the method for estimating it for a given stress concentration configuration will be discussed in the next section. Here, it is necessary to briefly introduce how to estimate the resistance curve for crack propagation, ΔK_th_.

Several methods for estimating the threshold curve [4,16,17,24] differ mainly in the hypotheses used for their derivation. The threshold for crack growth (resistance to crack propagation) increases from a minimum value, given by the intrinsic fatigue limit of the material, up to the value for long cracks, ΔK_thR_, which is independent of crack length for a given stress ratio *R*. This transition in terms of ΔK is modeled differently in each model. The recently proposed IBESS model [26] fits experimental data obtained for very short cracks to estimate the threshold as a power function of crack length, or proposed a variant of the El Haddad model. Alternatively, an exponential law has been suggested [16,24]. It should be noted that, despite their differences, any method can be employed to estimate the threshold curve as a function of crack length. However, previous works have demonstrated the advantages of using Chapetti’s model in several applications [34], particularly when dealing with crack lengths similar to the microstructural dimension, *d*, or when defining or estimating the minimum ΔK_th_ associated with the intrinsic fatigue limit, ΔK_dR_. A comparison of the main models is available in Ref. [33].

**Figure 6:**
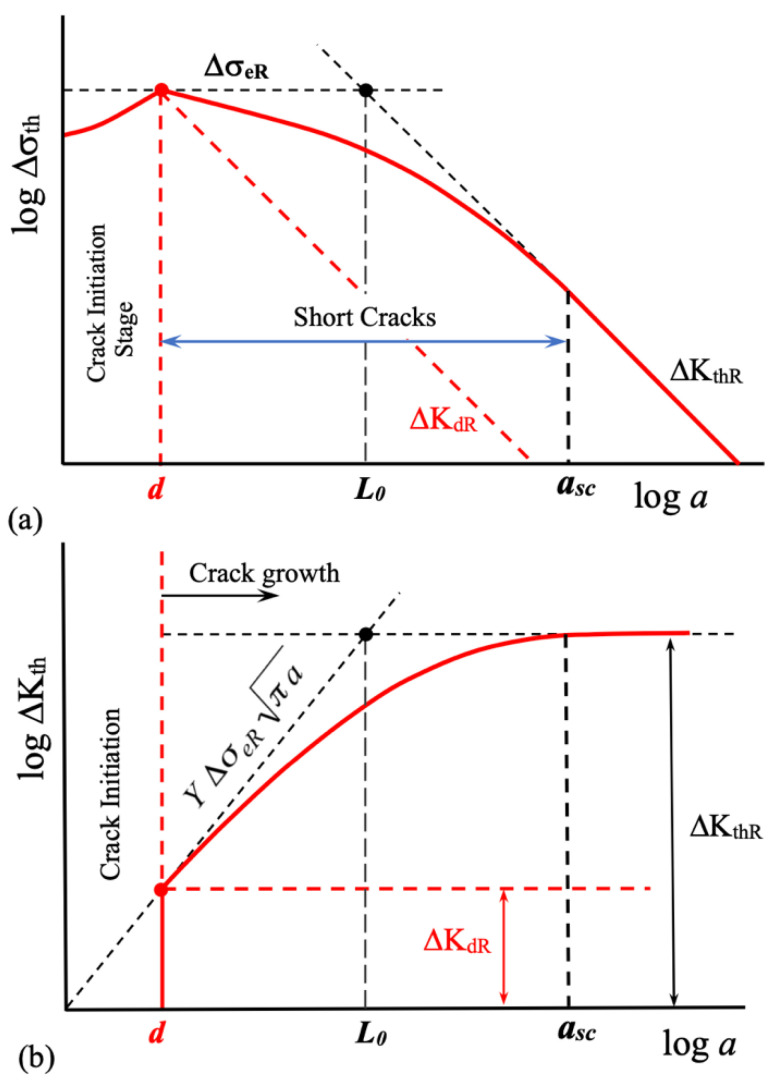
The fatigue crack propagation threshold, as defined by Equation (2), is presented in terms of (**a**) the stress range threshold, Δσ_th_ (Kitagawa–Takahashi diagram), and (**b**) the stress intensity factor range, ΔK_th_.

In the present work, the threshold for fatigue crack propagation as a function of crack length *a* is estimated with Equation (2), following Chapetti’s proposal [24]:

(2) $$\Delta K_{th}=\Delta K_{dR}+\left(\Delta K_{thR}-\Delta K_{dR}\right)\left[1-e^{k(a-d)}\right]$$

which is valid for *a* > *d*, with *d* being the average microstructural dimension (e.g., grain size, martensite lath size, etc.). Additionally, ΔK_dR_ represents the intrinsic (microstructural) fatigue threshold as given by Equation (3), *k* measures the development of the threshold as defined by Equation (4), and ΔK_thR_ is the long crack propagation threshold.

(3) $$\Delta K_{dR}=Y\Delta \sigma_{eR}\;\sqrt{\pi d}\;$$

(4) $$k=\frac{1}{4\;d}\;\frac{\Delta K_{dR}}{\left(\Delta K_{thR}-\Delta K_{dR}\right)}\;$$

where Δσ_eR_ is the intrinsic fatigue limit of the material. Figure 6 schematically illustrates the threshold given by Equation (2), shown in Figure 6a as a function of the stress range (Kitagawa–Takahashi diagram) and in Figure 6b as a function of the stress intensity factor range.

**Figure 7:**
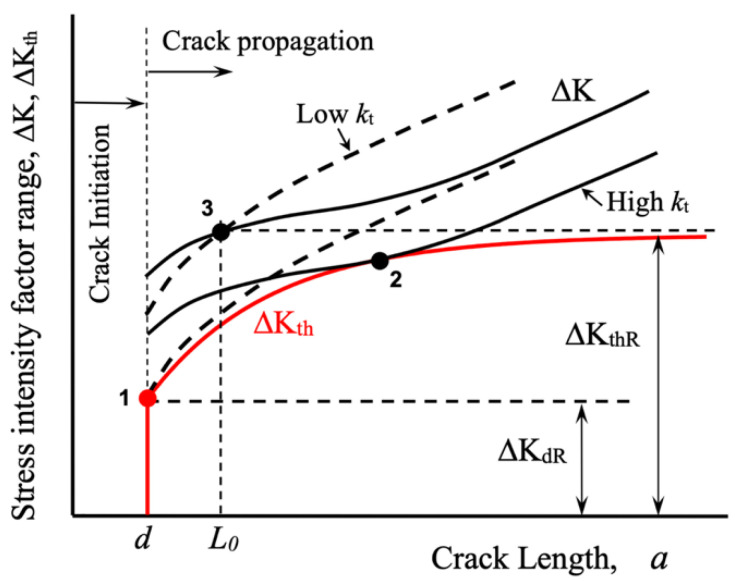
Schematic explanation of the threshold condition associated with the fatigue limit of a given notch configuration, in terms of the stress intensity factor range.

Figure 7 schematically depicts the threshold curve and the applied ΔK for two hypothetical stress concentrators, one blunt and the other sharp. A ΔK_th_ vs. *a* curve is also plotted (red line). As the stress concentration factor increases, the stress gradient near the notch root also increases, and the fatigue limit condition is associated with longer crack lengths (non-propagating cracks). In the diagram of Figure 7, for the blunt notch, the fatigue limit is associated with a crack length equal to the microstructural size *d*, similar to the case of the material’s intrinsic fatigue limit (point 1). In contrast, for a sharp notch, the stress gradient is such that the ΔK curve becomes tangent to the ΔK_th_ curve at longer crack lengths (point 2).

Point 3 in Figure 7 corresponds to the fatigue resistance to crack propagation associated with the point stress model approach (see also Figure 6). In this case, the threshold condition is defined for any configuration by the threshold for long cracks, ΔK_thR_ (a constant value for a given stress ratio *R*), and the intrinsic crack length *L*_0_ associated with the intrinsic fatigue limit, which is typically greater than *d*. Under this approach, the fatigue limit defined in this manner is associated with the same non-propagating crack for any stress concentration factor *k*_t_. In contrast, using the threshold curve approach, the threshold condition depends on the relative position of the applied driving force ΔK and the threshold for fatigue crack propagation ΔK_th_, both as functions of crack length. Consequently, the non-propagating crack associated with the fatigue limit (a threshold condition for fatigue crack propagation) depends on both curves (ΔK and ΔK_th_). In this scenario, as the stress concentration factor increases, the threshold condition gradually shifts from point 1 to point 2 as *k*_t_ increases, as depicted schematically in Figure 7. This analysis demonstrates the advantage of using a resistance curve to estimate the threshold condition for any configuration.

It is important to highlight that the fracture mechanics approach represented by Equation (1) gives rise to different proposed variants solely associated with the type of model used to estimate the threshold curve ΔK_th_ vs. *a*. The hypotheses used and the available data will determine which model best suits the desired estimations.

## 5. Estimation of the Applied ΔK for Cracks from Notches

Here, the estimation of ΔK applied to cracks nucleated and propagating from the root of blunt and sharp notches will be examined. It is important to consider that these cracks transition from being solely influenced by the stress field associated with the notch root to strongly depending on the depth *D* of the notch, a transition dictated by the geometry of the notch (depth *D* and root radius ρ).

Firstly, the estimation of ΔK will be mentioned for cases where the size of the concentrator cannot be quantified (many practical cases). Next, ΔK will be examined in the region influenced by the stress field associated with a notch, for which a depth dimension *D* can be defined, and ΔK for the case where the crack tip has moved beyond this field and is determined by the sum of the notch depth and the crack length (*D + a*). Finally, ΔK in the transition zone between these last two cases will be analyzed, and a simple expression will be proposed to estimate it. This approach allows for a comprehensive analysis using continuous expressions that define both the stress concentration effect and the notch size effect.

### 5.1. Integral Estimations of the Applied ΔK for General Configurations

In many practical applications, size effects are omitted because defining or measuring notch depth can be difficult or arbitrary. In such cases, the fracture mechanics approach can be applied in its general formulation, estimating the applied ΔK using methods such as finite element analysis (FEM). Here, the propagation threshold is considered from the surface at the hotspot where the crack nucleates and propagates.

In this scenario, several applications can be found in the literature. An example of such a case is the analysis of the fatigue behavior of welded joints [26,36], which has contributed to understanding the influence of factors such as welded plate thickness, local geometries, initial defect sizes, and undercuts. The case of undercuts [37] is particularly noteworthy as it combines geometric details of the welded joint, resulting in stress concentration, with the localized concentration created by the undercut acting similar to a notch with a specific depth *D* and root radius.

This case will not be analyzed in detail here, as the focus will be on well-defined notches with specified lengths and root radii, the effects of their sizes, and the limitations and transitions involved in reducing them to shallow notches or small volumetric defects.

### 5.2. Applied ΔK for Cracks in the Stress Field Associated to the Notch

Lukás [38] proposed the following expression to estimate the stress intensity factor range ΔK applied to cracks originating from the root of notches:

(5) $${∆K}_{BN}=\frac{Yk_{t}{∆\sigma }_{n}}{\sqrt{1-\frac{\alpha a}{\rho }}}\sqrt{\pi a}$$

where ρ is the notch radius, α is the stress gradient coefficient, *a* is the crack length from the notch root, and Δσ_n_ is the nominal applied stress range. The suffix BN is added to ΔK to indicate that it corresponds to a crack propagating through the stress field associated with the notch, and because it generally plays a role in defining the fatigue limit in the case of blunt notches. The factor *k*_t_/(1 − α *a*/ρ)^1/2^ represents the stress concentration introduced by the notch at a depth *a* from the notch root. α has been estimated as −2 [39], −4.5 [38], and −6 [40], among others. Reference [19] demonstrated that α = −4.5 provides a better fit and works reasonably well.

If we recall the concepts outlined in Figure 3 and Figure 4, from Equation (5) the stress concentration introduced by the notch at a given crack length depth a can be defined as follows:

(6) $$k_{ta}=\frac{k_{t}}{\sqrt{1+\frac{4.5\;a}{\rho }\;}}\;$$

Thus, the applied ΔK to a crack of length *a* will be given by the following:

(7) $${∆K}_{BN}=Y\;k_{ta}\;{∆\sigma }_{n}\;\sqrt{\pi \;a\;}$$

When the applied ΔK equals the threshold ΔK_th_, the applied nominal stress range Δσ_n_ will equal the fatigue limit of the notch, Δσ_e_.

Other expressions have been proposed by other authors, also analyzing the influence of the stress field associated with the concentrator to estimate the applied ΔK to a crack propagating from the root of such a notch. For example, Smith and Miller [3], Nisitani-Endo [8], Schijve [39], and Yates [41] have proposed alternative approaches. Here, the proposal by Lukas (Equation (7)) will be used. 

### 5.3. Applied ΔK for Cracks Tips Far from the Notch Stress Field

Once the crack propagates and leaves the singular stress field associated with the notch, the ΔK effectively applied to the crack becomes fundamentally influenced by the combined length of the crack and the notch depth, D. In this way, the applied ΔK can be estimated with the following expression:

(8) $${∆K}_{SN}=Y{∆\sigma }_{n}\sqrt{\pi \left(D+a\right)}$$

where Δσ_n_ is the nominal applied stress range for the configuration. Since notches are being dealt with, the hypothesis that the crack has developed a straight front, as observed in Figure 2, can be used. In this case, *Y* is taken as 1.12.

It is important to note that this approach can be applied without major issues to semi-circular, semi-elliptical, or similar notches, with an aspect ratio greater than or equal to one. For aspect ratios less than one (open notches), and to avoid greater complications, it is preferable to estimate the applied ΔK using the finite element method. It is also important to note that the parameter *Y* should additionally consider the finite dimensions of the analyzed geometric configurations and the load configuration, aspects that are not analyzed here but can be included in the estimations without difficulty.

### 5.4. Integral ΔK Solution for the Complete Crack Extension

**Figure 8:**
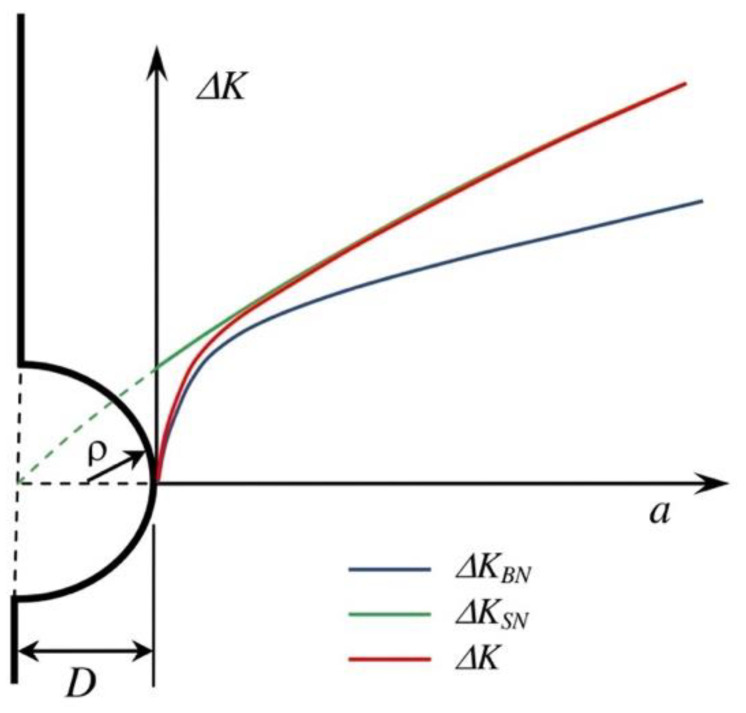
Schematic of the transition (red line) of the applied ΔK from Expression (7) to Expression (8).

The total transition is analyzed here as a continuum. Initially, the crack nucleates and propagates across the notch stress field, and the applied ΔK can be estimated by Equation (7). Subsequently, the applied ΔK becomes influenced by the size of the notch, and the transition occurs. Finally, the applied ΔK is determined by the effective crack length, (*D + a*) (Equation (8)). The actual crack driving force, ΔK, should gradually transition from Expression (7) to Expression (8), as shown schematically in Figure 8.

In order to obtain a unique function for the crack driving force ΔK, the following expression has been proposed [42]:

(9) $$∆K={∆K}_{BN}+\left({∆K}_{SN}-{∆K}_{BN}\right)\left[1-e^{-\frac{2}{\sqrt{D\rho }}\left(a-d\right)}\right]$$

where ΔK_BN_ and ΔK_SN_ are given by Expressions (7) and (8), respectively. *D* represents the notch depth, ρ is the notch ratio, and *d* is added to account for the minimum crack length that can be considered according to the definition of the intrinsic fatigue limit (position of the strongest microstructural barrier, see Figure 6 and Equation (3)). According to the proposed Expression (9), the transition is described by the function 2/(*D* ρ)^1/2^. This expression was chosen after an in-depth analysis of the various combinations of the main parameters controlling the transition (*D* and ρ) [42]. The numeral 2 was optimized following a parametric analysis using several values of *D* and ρ.

Furthermore, Meggiolaro et al. [43], after analyzing the behavior of cracks emanating from semi-elliptical notches, proposed the following numerical expression to estimate the applied ΔK. This expression accounts for both the notch stress field and the transition to the total crack length range:

(10) $$∆K=1.215{∆\sigma }_{n}\sqrt{\pi a}f\left(\frac{a}{D},\frac{\rho }{D}\right)$$

$$f=k_{t}\sqrt{\frac{1-\exp\left(-{k_{t}}^{2}\frac{a}{\left(D+a\right)}\right)}{{k_{t}}^{2}\frac{a}{\left(D+a\right)}}}$$

$$k_{t}=\left(1+2\sqrt{\frac{D}{\rho }}\right)\left(1+\frac{0.1215}{1+{\left(\frac{\rho }{D}\right)}^{2}}\right)$$

**Figure 9:**
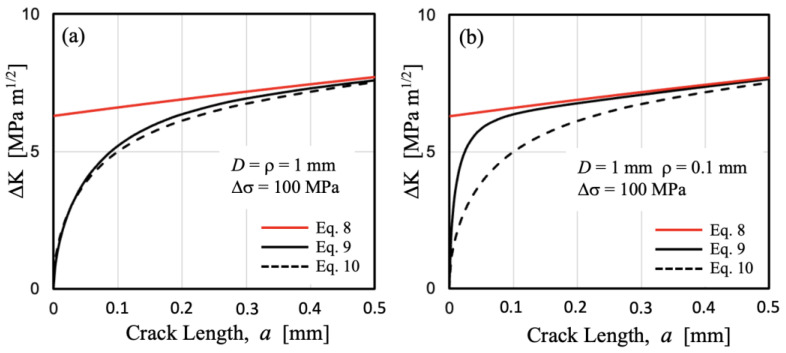
Applied ΔK given by Expressions (8)–(10) for a nominal stress range Δσ = 100 MPa and two different notches: (**a**) *D* = ρ = 1 mm, and (**b**) *D* = 1 mm, ρ = 0.1 mm.

Figure 9 compares the ΔK estimations given by Expressions (9) and (10) for cracks propagating from two notches with the same depth (*D* = 1 mm) and the same applied stress range (Δσ = 100 MPa), but different root radii: (a) ρ = 1 mm, and (b) ρ = 0.1 mm. It can be observed that for the semi-circular notch (Figure 9a), Expressions (9) and (10) estimate similar values of ΔK vs. *a* over the entire transition range. However, for the case of the 0.1 mm root radius, a clearly sharper notch with a *k*_t_ of approximately seven, the transition is markedly faster according to Expression (9), making it more conservative when estimating the fatigue limit.

It is important to note that there are previous indications of (*D* ρ)^1/2^ controlling the transitions, although the physical mechanism of this transition in defining fatigue resistance has not been fully clarified. For example, early work by Smith and Miller [44] showed that the extent of the stress field was equal to 0.13 times (*D* ρ)^1/2^. More recently, the work of Yates [41] proposed the following expression to estimate the transition between the solutions given by Expressions (7) and (8), indicated by the parameter *F*:

(11) $$\frac{∆K}{{∆K}_{SN}}\approx F=4.012{\left(\frac{a}{\sqrt{D\rho }}\right)}^{0.5}-5.244\left(\frac{a}{\sqrt{D\rho }}\right)+2.245{\left(\frac{a}{\sqrt{D\rho }}\right)}^{1.5}$$

**Figure 10:**
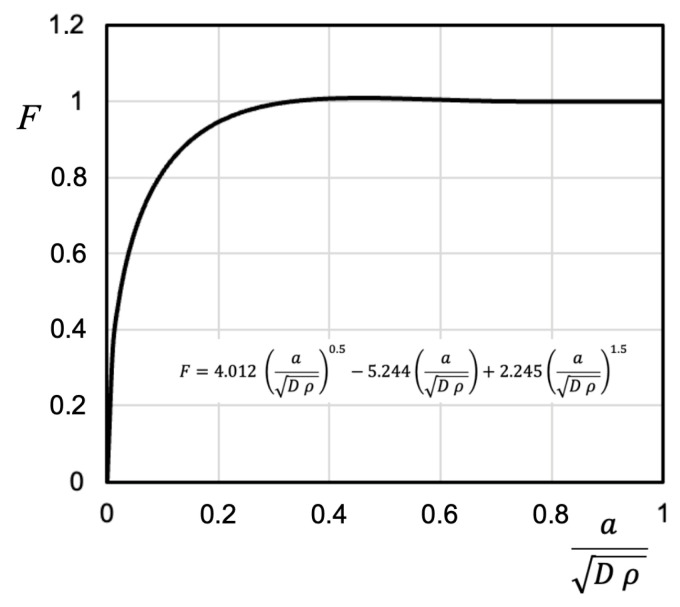
Transition parameter proposed by Yates [41].

The transition is completed when the parameter *F* equals one, meaning the applied ΔK becomes equal to that given by Expression (8). Figure 10 shows the evolution of this relationship with the non-dimensional distance *a*/(*D* ρ)^1/2^. Yates concludes that the applied ΔK reaches that given by Expression (8) when this parameter equals 0.4. However, it can be seen that the parameter *F* exceeds a value of 0.95, indicating a difference of less than 5% between the two solutions, starting from *a*/(*D* ρ)^1/2^ ≈ 0.2.

Analyzing the configurations in Figure 9, the parameter *a*/(*D* ρ)^1/2^ = 0.2 yields values of *a* = 0.2 mm for case (a) and 0.063 mm for case (b). By examining Figure 9, it can be inferred that the results indicate that Expression (9) predicts acceptable transitions for the applied ΔK value.

The results also show that the parameter used, *a*/(*D* ρ)^1/2^, is appropriate for describing the transition, and will help us define and/or estimate a non-propagating crack length for the case of sharp notches.

## 6. Fatigue Notch Sensitivity and Size Effect

Here, the application of the fracture mechanics approach for the integral estimations of the fatigue notch sensitivity and size effect will be analyzed, especially for configurations where the notch size can clearly be defined.

It is important to note that the fracture mechanics approach can be applied to any component under high-cycle fatigue, even for complex configurations for which no analytical expressions exist to estimate the applied ΔK, or no simplifications can be defined to provide them. In these cases, the applied ΔK should be estimated using finite element models. Although the application of this methodology is complex and requires specialized expertise, it allows for the analysis of the influence of most variables involved in defining the fatigue limit of the analyzed component. Undoubtedly, this approach will eventually be implemented in design recommendations following the development of conservative simplifications that make it applicable by engineers with basic knowledge of fracture mechanics.

### 6.1. Simplified Fracture Mechanics Approach and Hypotheses

The size effect is evident when dealing with semi-circular or elliptical notches with the long axis perpendicular to the loading direction. In this case, the notch size influences the transition of the applied ΔK analyzed in the previous section.

**Figure 11:**
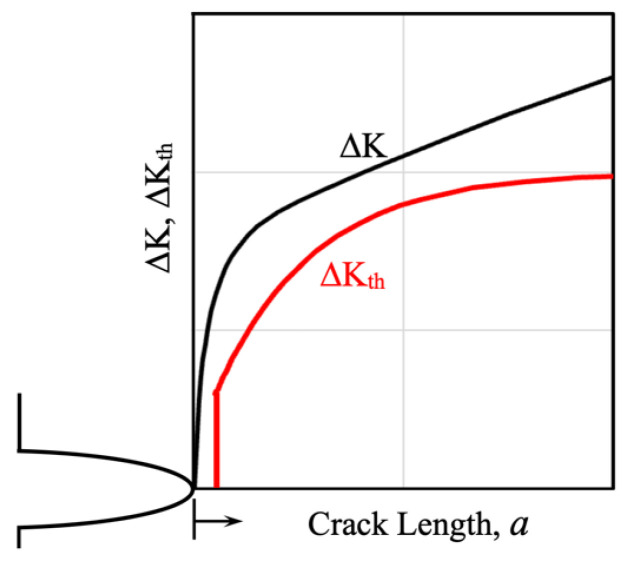
Schematic representation of the configuration for analyzing notches using the fracture mechanics approach.

Here, it is important to emphasize the need to define a hypothesis regarding the resistance curve ΔK_th_ vs. *a*, as the propagation threshold depends on the crack length in the early propagation stage. Additionally, the system “sees” a crack length that transitions from the length measured from the notch root to a length that includes the notch depth *D*, as discussed in the previous section. The hypothesis that the propagation threshold depends solely on the crack length measured from the surface (root) of the notch, as indicated in Figure 11, will be used. This hypothesis is justified by Maierhofer et al. [25] in terms of the development of crack closure as the sole effect on the increase in the propagation threshold ΔK_th_. They indicate that the crack flanks can be in contact only over this length, that the notch depth *D* is not subjected to any crack closure even under compression loading, and that only by the crack extension from the notch root the build-up of crack closure is possible. Therefore, we will use the hypothesis that the development of the threshold curve as a function of crack length begins from the root of the notch.

The fatigue threshold condition that defines the fatigue resistance of the configuration for any notch geometry and size can then be estimated by comparing, according to Equation (1) and Figure 5, the crack driving force ΔK given by Equation (9) and the threshold for fatigue crack propagation as a function of crack length that can be estimated using Equation (2).

To estimate the propagation threshold, the Chapetti model introduced in Section 4 (Equation (2)) will be used. However, other models can be used, such as the El Haddad model [4], or those that only use the concept of crack closure, like the models proposed by McEvily and Minakawa [16], Tanaka and Akiniwa [17], or Kolitsch et al. [45]. Regarding the proposal by Kolitsch and his collaborators, it is important to note that in the work published in reference [25], Maierhofer et al. applied their proposal to analyze finite notch depths and compared it with the predictions given by the El Haddad model, considering for the latter that the crack length includes the notch depth *D*. In this way, two models using different hypotheses were compared. If the same hypothesis were used when applying the El Haddad model, similar results would be obtained, only differentiated by the threshold estimation as a function of the crack length provided by each model. Additionally, and following the same criteria, they erroneously indicate that the Chapetti model neglects the influence of the depth of a pre-existing flaw and that it leads to non-conservative predictions. Recently, the same error has been repeated by Roveda et al. [46], reiterating what was stated by Maierhoher et al. [25] about the El Haddad model and the Chapetti model. Furthermore, when the influence of voids in AlSi10Mg fabricated by PBF-LB/M was analyzed, both the approach proposed by Maierhofer et al. [25] and the cyclic R-curve method proposed by Tanaka and Akiniwa [17] were applied (the latter being used by Chapetti in all applications of his model given by Equation (2)). They concluded that both methodologies predict similar fatigue limits and are more conservative than the application of the El Haddad model. If the same hypothesis were applied to the El Haddad model using either methodology, an even more conservative prediction could potentially result, particularly for the analyzed void size regime (smaller than 0.1 mm).

It is important here to emphasize that models proposed to predict the threshold curve, ΔK_th_ vs. *a*, should not be confused with the hypotheses used for applying the fracture mechanics approach to estimate fatigue limits and fatigue lives of a given configuration. The application will require suitable hypotheses, as in the case of notches or notch-like defects. In other words, it is necessary to clarify that it is not an issue with the models used, but rather an analysis of the two possible hypotheses: whether or not to include the depth of the notch or defect in the length associated with threshold development.

It is clear, at least to the present author, that for relatively large notches, the depth of the notch should not be included, and it is pertinent and even conservative to assume that the threshold develops from the root of the notch or defect. However, for small notches or defects, this is not clear, even when analyzing in detail the results of Maierhofer et al. [25]. This issue will be revisited in Section 7, where the case of small notches or defects, from which similarly sized three-dimensional cracks are generated, will be analyzed. A different hypothesis could be used for this case. Due to the orders of magnitude changes in some variables, substantial variations arise in the considerations that need to be taken into account when formulating the hypotheses associated with the fracture mechanics approach.

### 6.2. Applications

To demonstrate the approach’s ability to estimate the fatigue notch sensitivity and size effect in fatigue, three sets of experimental results taken from references [21,47,48] are analyzed by applying the configuration shown in Figure 11, the fracture mechanics approach illustrated in Figure 5 and given by Equation (1), as well as Expressions (2) and (9) to estimate the threshold for crack propagation, ΔK_th_, and the applied ΔK, respectively. The data used for estimations are shown in Table 1. For a given notch depth *D*, the stress concentration factor is altered by changing the notch radius ρ according to the expressions provided by the analyzed references for semi-elliptical notches.

**Figure 12:**
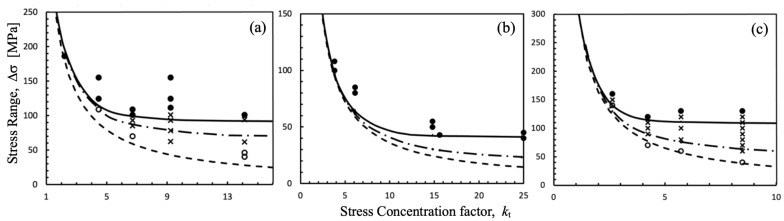
Experimental data from references and estimations by using the fracture mechanics approach (Equations (1), (2) and (9)). (**a**) 0.22C steel [47], (**b**) C10 Steel [21], and (**c**) SM41B steel [48]. Full circles indicate fracture, hollow circles indicate no fracture without non-propagating cracks, and crosses indicate no fracture with non-propagating cracks in specimens.

Experimental results provided by references and estimations made using the proposed approach are shown in Figure 12a–c, respectively. Full circles indicate fracture, hollow circles indicate no fracture without non-propagating cracks, and crosses indicate no fracture with non-propagating cracks in specimens. In all figures, three estimation curves are shown, corresponding to *k*_t_, *k*_td_ (first barrier, placed at *d* from the surface), and *k*_f_. The dashed lines are given by Δσ_e_ = Δσ_eR_/*k*_t_, the dashed-dotted lines are given by Δσ_e_ = Δσ_eR_/*k*_td_ (with *k*_td_ given by Equation (6) and the position from the surface of the first and the strongest microstructural barrier, *d*), and the solid lines are estimated by applying the integral fracture mechanics approach and comparing the applied ΔK given by Expression (9) and the threshold ΔK_th_ estimated by Expression (2) using the configuration shown in Figure 11. For each *k*_t_, the fatigue limit of the configuration is estimated as the nominal stress level for which the applied ΔK is equal to ΔK_th_ at a given crack length, which represents the non-propagating crack associated with the configuration (schematic in Figure 5b or point 2 in Figure 7).

Figure 12 demonstrates excellent predictions, not only in estimating the level of fatigue resistance but also in capturing the transition from blunt to sharp notches. It is important to highlight that the results are truly estimated, as the procedure does not require any fitting parameters.

### 6.3. Simple Estimation of the Maximum Fatigue Notch Factor, k_f_^Max^

**Figure 13:**
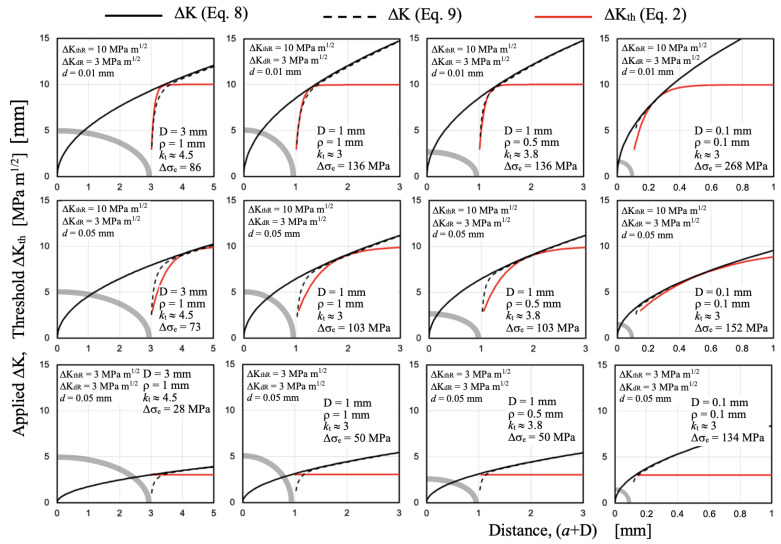
Configurations corresponding to the fatigue limit, Δσ_e_, of different geometric setups for a hypothetical material: ΔK_dR_ = 3 MPa√m, ΔK_thR_ = 10 MPa√m, and two microstructural sizes *d*: 0.01 mm and 0.05 mm. Gray thick line describes the notch shape.

This section of the paper will describe a simple way of estimating the minimum fatigue limit associated with a specific notch size, which will be determined by high values of *k*_t_ generated by small root radius values, ρ. For this, it is necessary to analyze the non-propagating crack length associated with the configuration that defines the fatigue limit. Figure 13 shows curves of ΔK and ΔK_th_ as a function of the distance from the notch root surface, corresponding to the fatigue limit Δσ_e_ of different notches and for a hypothetical material (ΔK_dR_ = 3 MPa√m, ΔK_thR_ = 10 MPa√m), and two microstructural sizes *d* (0.01 mm and 0.05 mm). The geometric configurations correspond to different combinations of notch depth *D* (0.1 mm, 1 mm, and 3 mm) and root radius ρ (0.1, 0.5, and 1 mm). The propagation threshold ΔK_th_ vs. *a* is estimated using Equation (2) and develops from the root of the notch, according to the hypothesis used (red lines). The solution given by Equation (8) (solution that includes the notch depth, *D*) is shown with a solid black line, and the ΔK estimated by Equation (9) (integral solution for a crack starting from the root of the notch) is shown with a dashed black line. The configurations in the bottom row of graphs consider the case of high load ratios *R*, for which the long crack threshold approaches the minimum values measured experimentally (assumed to be 3 MPa√m for this case).

From the results obtained, the following important observations can be inferred:

- The range of crack lengths where the applied ΔK is influenced by the stress field associated with the stress concentrator generally does not coincide with the non-propagating crack length associated with the fatigue limit of the configuration. It is evident that the threshold curve and its development are crucial in defining the configuration associated with the fatigue limit.
- In almost all cases, the applied ΔK estimated by the simple Equation (8) allows for the estimation of the non-propagating crack length associated with the fatigue limit. In cases where differences are observed with the ΔK indicated by Equation (9), Equation (8) provides conservative values for fatigue limit estimations.
- For notch depth *D* above a certain value, D*, the fatigue limit would be determined by the threshold for long crack propagation. This value D* increases with increasing microstructural size *d*, due to the increased range of short cracks (the threshold fully develops at longer lengths). Conversely, D* decreases with decreasing long crack threshold ΔK_thR_, and hence, with increasing load ratio *R*.
- For the same geometric configuration, an increase in microstructural size *d* expands the range of short cracks, reduces the fatigue limit of the configuration, and increases the associated non-propagating crack length.
- For relatively small notch sizes (right column of graphs in Figure 13, where *D* = ρ = 0.1 mm), the hypothesis used and the results obtained indicate that the non-propagating crack associated with the fatigue limit would take values similar to or greater than the notch depth *D*. Its length would significantly increase with increasing microstructural size *d*. The case of small notches will be analyzed in more detail in the next section.

An important point to emphasize here is that, according to these observations, it can be concluded that the non-propagating crack length associated with the fatigue limit of sharp notches is primarily determined, in most cases and especially for notch depths greater than about 0.5 mm, by the range of short cracks. While in some cases the lengths are slightly shorter, they generally exceed half of this range, making it conservative to consider this fully and encompass all cases. It would therefore be important to estimate the range of short cracks, that is, the range of lengths over which the threshold ΔK_th_ develops.

**Figure 14:**
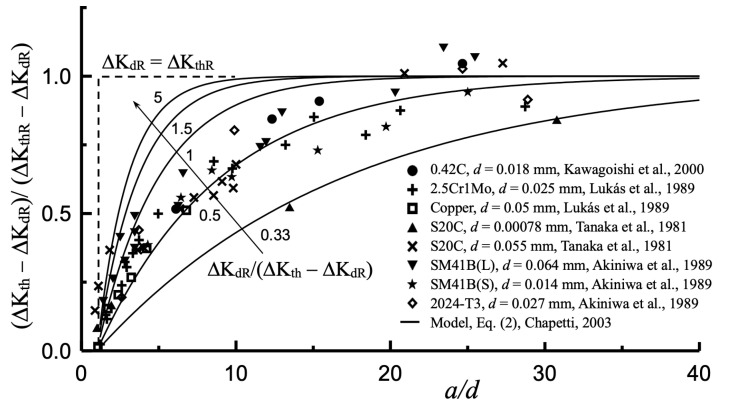
Development of the fatigue crack propagation threshold as a function of non-dimensional crack length [14,15,24,49,50]. Used with permission, Elsevier, LN 5873120046227.

Figure 14 presents the experimental results of the thresholds compiled in [24] when proposing the prediction model given by Equation (2), in terms of the development of the component (ΔK_th_ − ΔK_dR_) as a function of the non-dimensional crack length scaled by the microstructural size *d*. The estimations given by Equation (2) for different ratios ΔK_dR_/(ΔK_thR_ − ΔK_dR_) are also shown. It can be observed that in most cases analyzed, the threshold develops fully for a crack length of approximately 20 times the microstructural size (*a*/*d* = 20). This observation can then be used to estimate the non-propagating crack length associated with the fatigue limit of sharp notches, such that:

(12) $$a_{np}\approx 20d$$

It is necessary to clarify here that, as seen when analyzing Figure 13, as *R* increases, the propagation threshold decreases and the range of short cracks becomes smaller, so this estimation would be conservative, and conservatism would increase with increasing *R*.

Using Equation (8), replacing the crack length with the non-propagating crack length associated with the fatigue limit, and equating the applied ΔK to the threshold for long crack propagation, we can write the following:

(13) $$∆K=Y{{∆\sigma }_{e}}^{Min}\sqrt{\pi \left(D+a_{np}\right)}={∆K}_{thR}$$

where Δσ_e_^Min^ corresponds to the minimum fatigue limit associated with sharp notches. Solving for the fatigue limit, assuming that the non-propagating crack length can be estimated as 20 times the microstructural size *d* (*a*_np_ = 20 *d*), we obtain:

(14) $${{∆\sigma }_{e}}^{Min}=\frac{{∆K}_{thR}}{Y\sqrt{\pi \left(D+20d\right)}}$$

For any sharp notch configuration with high stress concentration factors (*k*_t_), it can be further assumed that non-propagating cracks develop a straight crack front, as illustrated in Figure 2d.

By dividing the the intrinsic fatigue limit of the material, Δσ_eR_, by the minimum fatigue limit associated with the notch depth *D*, Δσ_e_^Min^, the maximum fatigue notch factor, *k*_f_^Max^, is obtained as follow:

(15) $${k_{f}}^{Max}=\frac{{∆\sigma }_{eR}}{{{∆\sigma }_{e}}^{Min}}$$

Applying Equation (14) to the results reported by Tanaka et al. [48] for SM41B steel (see Table 1), we obtain a minimum fatigue limit Δσ_e_^Min^ = 95 MPa, 20% lower than the one obtained experimentally for *k*_t_ = 4.23. For the data reported by Meneguetti et al. [21] corresponding to C10 steel (see Table 1), Equation (14) estimates a minimum fatigue limit Δσ_e_^Min^ = 38 MPa, only 5% below the experimental measurement for *k*_t_ = 25.

It is important to emphasize that the analyses and results allow us to infer the importance of the resistance curve in defining the notch size effect on the fatigue limit. Expression (14) indicates that in determining the minimum fatigue limit of the configuration, not only the size (depth) of the notch *D* is involved, but also the microstructural size, *d*, and the threshold for long cracks, ΔK_thR_.

## 7. Fatigue Limit of Small Notches

Up to this point, it can be stated that for relatively large notches or defects, it is necessary to consider the hypothesis illustrated in Figure 11, that is, to assume that the propagation threshold ΔK_th_ develops from the root of the notch or defect. However, for small notches or defects, this is not clear. To analyze this case, we will use the experimental data reported by Nisitani and Endo [8] on the fatigue strength of 0.45C steel specimens with the following properties: yield stress σ_y_ = 364 MPa, ultimate tensile strength σ_u_ = 632 MPa, intrinsic fatigue limit Δσ_eR_ = 560 MPa, and mean ferrite size *d* = 0.006 mm. In particular, the reports corresponding to the fatigue strength of a component with a notch depth *D* = 0.1 mm and a notch radius ρ = 0.02 mm will be used. For this case, the fatigue limit experimentally obtained is Δσ_e_ = 360 MPa, and the observed non-propagating crack length was similar to the microstructural size *d*, meaning that the fatigue limit in this case is defined by the microstructural threshold ΔK_dR_.

To perform the analysis, the value of the threshold for long crack propagation, ΔK_thR_, is also needed, though it was not reported by Nisitani-Endo. However, it can be estimated from the data reported for a deeper and sharper notch and applying Equation (14), with dimensions *D* = 1.5 mm and ρ = 0.02 mm. To apply Equation (14) for this particular case to estimate the threshold for long cracks ΔK_th_ (knowing Δσ_e_), the parameter *Y* must first be calculated for the geometrical and loading configuration used by Nisitani and Endo (bending loading and round bar samples with a diameter of 8 mm and a notch depth *D* = 1.5 mm). A value of 0.45 is obtained from the *Y* solution reported in reference [51] for a crack length of (*D + a*) = 1.62 mm. Then, by applying Equation (14), a value of ΔK_thR_ = 8 MPa√m is obtained, which is acceptable for the strength of the studied steel and its relatively small microstructural size.

**Figure 15:**
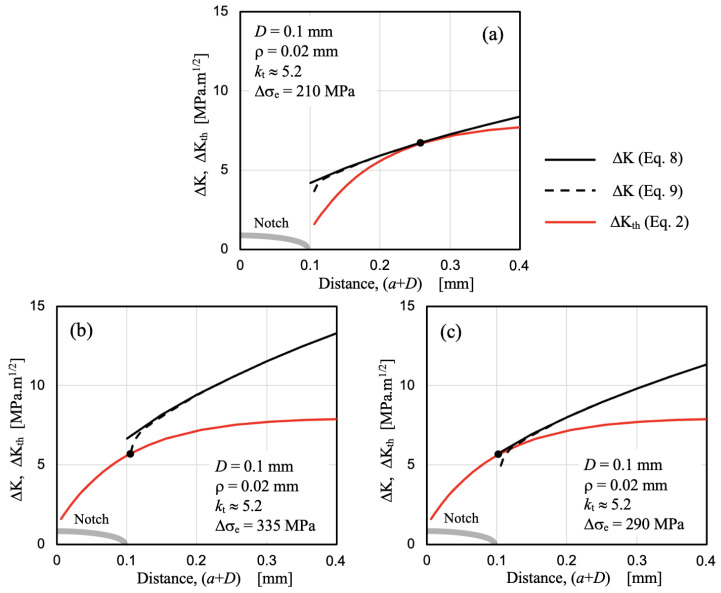
Estimations of the applied ΔK (Equations (8) and (9)) and the threshold ΔK_th_ (Equation (2)) are provided for a small notch with *D* = 0.1 mm and ρ = 0.02 mm. The nominal applied stress Δσ corresponds to the estimated fatigue limit of the notch, Δσ_e_. (**a**) The fatigue limit is estimated by assuming that the threshold develops from the notch root; the same results are given by both Equations (8) and (9). (**b**) The fatigue limit is estimated using Equation (7), including the notch depth *D* in the development of the threshold. (**c**) The same as (**b**), but Equation (8) is used. Gray thick line describes the notch shape.

Three configurations analyzed for the case of the small notch with *D* = 0.1 mm and ρ = 0.02 mm are shown in Figure 15a–c. In Figure 15a, the hypothesis illustrated in Figure 11 is applied, resulting in a fatigue limit Δσ_e_ = 210 MPa and a non-propagating crack length of about 0.16 mm. Figure 15b and Figure 15c illustrate two cases where the crack length used for the development of the propagation threshold ΔK_th_ includes the notch depth (red lines). In Figure 15b, the applied ΔK is estimated using Equation (9), meaning the applied ΔK is estimated for a crack initiated from the root of the notch and influenced by its associated stress field. For this case, the fatigue limit obtained is Δσ_e_ = 335 MPa, and the associated non-propagating crack is determined by the microstructural size *d*. Figure 15c shows the configuration that allows estimating the fatigue limit considering the simplified Equation (8), which includes the effective length given by (*a + D*). For this case, the estimated fatigue limit is Δσ_e_ = 290 MPa, slightly lower than the previous case as expected (conservative), with a non-propagating crack similar in size to the microstructural size *d*.

The results obtained clearly show that the configuration considered in Figure 15b provides an acceptable estimation, 7% smaller than the value experimentally measured by Nisitani-Endo for the notch considered (Δσ_e_ = 360 MPa), as well as a similar non-propagating crack length, *a*_np_ = *d*. The case presented in Figure 15c is conservative by 20%, resulting from assuming the notch as a crack of length (*a + D*). The approach used for relatively large notches (as in previous sections), as indicated in Figure 15a, yields a fatigue limit value that is 42% smaller, indicating a very conservative estimation in this case. In addition, this configuration estimates a non-propagating crack length of about 0.16 mm, too long when compared to the length observed by Nisitani-Endo, similar to the microstructural dimension *d*.

The use of the configuration that considers the threshold developed from the surface (including the small notch or defect) often requires the need to estimate the intrinsic material strength. This becomes evident in cases where the material has inherent defects larger than the microstructural size *d*. In such cases, the Murakami–Endo model [52] is commonly used to describe the influence of defects and estimate how their size increase decreases the material’s fatigue limit. The application of this model implicitly involves the hypothesis used in Figure 15c including the defect size in the development of the threshold curve ΔK_th_ vs. *a* (or Δσ_th_ vs. *a*).

It is important to note that the Murakami–Endo model implicitly has a limitation in its application to defect sizes smaller than the microstructural size, sometimes overlooked when analyzing the influence of defects on the fatigue limit of metal alloys. This lower limit must be defined by estimating the intrinsic strength of the defect-free material, for which it is necessary to develop predictive models of acceptable reliability. Estimating this intrinsic fatigue limit Δσ_eR_ (or the associated ΔK_dR_) from simple expressions that use the static or yield strength of the material involves significant uncertainties that can substantially reduce the reliability of the analyses performed. A more detailed analysis on this topic can be found in reference [53].

## 8. Discussions and Remarks

**Figure 16:**
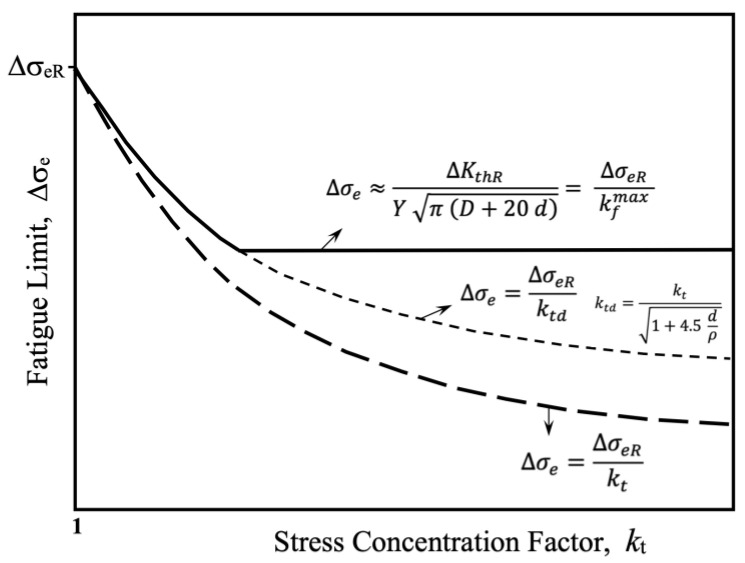
Schematic representation of the three important curves associated with the Frost diagram. Fatigue limits for a specific notch depth *D* are plotted as a function of the stress concentration factor *k*_t_.

Figure 16 schematically represents three important curves associated with the Frost diagram, where fatigue limits associated for a specific notch depth *D* are plotted as a function of stress concentration factor *k*_t_ (which increases as the notch radius ρ decreases). The long-dashed line indicates nominal stress levels required to generate stresses at the root surface of the notch similar to the intrinsic fatigue limit, given by the expression Δσ_eR_/*k*_t_. The short-dashed line, given by Δσ_eR_/*k*_td_, indicates nominal stress levels to generate stresses similar to the intrinsic fatigue limit at a depth *d* from the root surface of the notch (average microstructural dimension). The solid black line indicates the fatigue limit for all *k*_t_. The segment where this limit decreases with *k*_t_ defines the range of blunt notches, given by *k*_td_, while the horizontal segment defines the minimum fatigue limit associated with depth *D*, given by Δσ_eR_/*k*_f_^Max^. The *k*_f_^Max^, given by Equation (15), allows for estimating the minimum fatigue limit for sharp notches, defined by notch size *D*, the threshold for long crack propagation ΔK_thR_ for the applied load ratio *R*, and the microstructural size *d* (Equation (14)). These latter two values also define the non-propagating crack associated with the fatigue limit, along with the intrinsic fatigue limit, Δσ_eR_. In the range of sharp notches, where the fatigue limit becomes approximately independent of *k*_t_, the associated non-propagating crack has dimensions larger than the microstructural size *d*, and can reach lengths up to 20 times *d*. 

While for the case of blunt notches non-propagating cracks also develop, their length is much shorter and could conservatively be estimated with the microstructural size *d*.

The proposal of Equation (14) represents a significant technological contribution, as it enables the inference of the threshold for long cracks, ΔK_thR_, from measurements of the fatigue limit, Δσ_e_, in configurations with a sharp notch (high *D* and small ρ). Future experimental validations of Equation (14) across various materials and configurations will be crucial for confirming its applicability and accuracy.

It is important to clarify that Figure 16 shows a simplification of the indicated concepts. The comprehensive application of the analyzed fracture mechanics methodology allows a continuous Δσ_e_ vs. *k*_t_ curve to be estimated, with a smooth transition between the two solutions (blunt and sharp notches).

For relatively very small notches, with depths less than 10–20 times the microstructural size *d*, the hypothesis used for large notches, that the threshold curve should be considered from the root of the notch, seems not to work. Instead, the hypothesis that the size of the small notch or notch-like defect can be included in the crack length used to quantify the development of the ΔK_th_ threshold appears to work. This hypothesis aligns with the one implicitly used in most documented fracture mechanics analyses studying the influence of different types of defects on the fatigue limit of metallic alloys, including numerous applications of the Murakami–Endo model. Additional simplifications can be obtained by simplifying the shape of the defect or notch considered, which should be conservative.

However, it is necessary to consider that above a certain size, this approach will not be conservative. In the analysis of defect size distributions that include both size ranges, the transition between both approaches must be considered, although the expected larger defects in the analyzed case will define which approach is more conservative. The literature includes analyses, for example, of pore size distributions that are sometimes considerable, where the conservative approach will clearly be that of large concentrators, i.e., considering that the propagation threshold develops from the root of the notch (Figure 11). For intermediate sizes, it will be necessary to analyze the transition between hypotheses and define the most suitable one for the analyzed configuration.

Another important factor to consider is the development of the aspect ratio of the crack. In cases with small defects or notch-like defects, the aspect ratio will be clearly defined by the loading configuration. However, for relatively large notches with aspect ratios closer to zero (where the surface length is large compared to its depth), the aspect ratio of the crack developed from the notch root will quickly develop towards values close to zero, with a straight or nearly straight front. Three-dimensional cases with aspect ratios close to one (such as pores, pits, etc.) present a more complex scenario, as the evolution of the crack aspect ratio will be clearly influenced by the loading configuration (stress gradients). The hypothesis to be used regarding the propagation threshold development will depend on the size of the three-dimensional defect or notch, with transitions expected to larger sizes due to the three-dimensional nature of the configuration.

It is important to emphasize that all this analysis requires extensive further study, especially experimental, dealing with naturally nucleated small cracks. The identification of propagation thresholds and the appropriate hypothesis for the geometry–load–material configuration studied are crucial to predict fatigue behaviors and reliable fracture limit levels.

Finally, it is important to note that fatigue cracks often initiate from notches under conditions of local plasticity. However, it should be noted that when analyzing the fatigue limit of a given configuration, the notch field is typically elastic, especially for blunt notches. The maximum stress at the notch root is similar to the plain fatigue limit when the notch is blunt, which is generally lower than the yield stress of the material. As the notch becomes sharper (resulting in high stress concentration), it may lead to stress levels at the surface higher than the plain fatigue limit. In such cases, the fatigue limit is determined by a threshold condition associated with a non-propagating crack, where the potential plastic zone associated with the notch is confined behind the non-propagating crack tip. The non-propagating crack tip associated with the fatigue limit (a threshold condition) remains within the elastic stress field of the notch. This is the scenario studied in this work. For fatigue behavior analysis of notched components subjected to higher stress levels that induce significant plastic zones at the notch root, a more complex plastic notch field involving residual stresses, cyclic hardening, or softening should be considered.

## 9. Conclusions

This paper applies an integrated fracture mechanics approach to analyze and address both fatigue notch sensitivity and the notch size effect. It allows for advancing our fundamental understanding of the factors influencing these phenomena and for proposing straightforward tools for handling fatigue notch sensitivity and the notch size effect. The analysis incorporates considerations of notch sharpness, notch size, and material properties to account for the fatigue notch factor *k*_f_ across blunt and sharp notches. 

The following conclusions can be drawn:

- The fracture mechanics method that uses the ΔK_th_ vs. *a* resistance curve over the entire propagation range (from a depth equal to the microstructural size) allows both the fatigue limit of a notched component and the associated non-propagating crack length to be estimated.
- The applications of the methodology to documented cases in the literature show acceptable results for predicting the fatigue limit as a function of the stress concentrator *k*_t_.
- Non-propagating cracks are generated over the entire range of *k*_t_, even for blunt notches. Their length initially matches the microstructural size for *k*_t_ = 1 (smooth surface) and progressively increases as *k*_t_ increases. The maximum possible length would be determined by the size of the short crack range, within which the propagation threshold develops.
- An estimation of the short crack range is proposed, given by approximately 20 times the microstructural size *d*.
- A simple formula is proposed to estimate the maximum notch fatigue factor *k*_f_^Max^ for predicting the minimum fatigue limit for a given notch depth *D*.
- It is shown that the hypothesis generally used for relatively large notches, where their size is not included in the crack length calculation, would not be suitable for small notches or volumetric defects. For the latter case, it is necessary to generate more and better experimental evidence to develop appropriate hypotheses.

## Figures and Tables

**Table 1:** Experimental data for estimations shown in Figure 12.

**Ref.**	**Material**	***R***	**Δσ_eR_** **[MPa]**	**ΔK_thR_** **[MPa m^1/2^]**	***d*** **[mm]**	***D*** **[mm]**	**ρ** **[mm]**	***k*_t_**
[47]	0.22C Steel	−1	395	13	0.068	5.08	1.27	2.2
							0.5	4.47
							0.25	6.71
							0.1	9.23
[21]	C10 Steel	0.1	360	5.6	0.018	5	3	3.8
							1	6.1
							0.145	14.8
							0.13	15.6
							0.05	25
[48]	SM41B Steel	−1	326	12.36	0.064	3	3	2.63
							0.83	4.23
							0.39	5.72
							0.16	8.48

## Data Availability

The original contributions presented in the study are included in the article, further inquiries can be directed to the corresponding author.
